# Cardiac Tamponade in an 18-year-old Male with Undiagnosed Systemic Lupus Erythematosus

**DOI:** 10.7759/cureus.5186

**Published:** 2019-07-21

**Authors:** Neil P Larson, Thomas C Frawley, Brit Long

**Affiliations:** 1 Emergency Medicine, Brooke Army Medical Center, Fort Sam Houston, USA

**Keywords:** systemic lupus erythematosus, cardiac tamponade, pericardial effusion

## Abstract

Systemic lupus erythematosus (SLE) is a multisystem, autoimmune condition of extremely variable presentation and prognosis. While pericardial effusion is a common disease sequela, subsequent tamponade is a rare, potentially fatal complication. We present the case of an 18-year-old, previously healthy male who presented to the emergency department with a chief complaint of abdominal pain and hematochezia. Workup revealed massive pericardial effusion with tamponade pathophysiology requiring emergent pericardiocentesis, with further workup confirming a diagnosis of SLE. While SLE often presents in an indolent manner, cardiac tamponade may be the initial presentation of this disease.

## Introduction

Systemic lupus erythematosus (SLE) is an autoimmune disease affecting multiple organ systems with an extremely variable phenotype and prognosis. SLE has a much higher prevalence in females than males, with an affected female to male ratio as high as 15:1 [1]. Disease manifestations, such as joint disease, cutaneous and mucosal lesions, serositis, renal injury, and neurological manifestations, may occur. Cytopenias and other immunological laboratory abnormalities may also be present during the disease course. These sets of disease manifestations and laboratory abnormalities are both utilized in diagnosis for the 1997 American College of Rheumatology (ACR) SLE Classification Criteria and the 2012 Systemic Lupus International Collaborating Clinics (SLICC) Criteria, as well as more recently proposed SLE diagnostic criteria by the ACR and the European League Against Rheumatism (EULAR) presented in 2017 [2-3]. While pericardial effusion is common in SLE patients with a prevalence up to 50%, cardiac tamponade is a rare and potentially lethal complication, estimated to occur in less than 1% of those affected with SLE [4]. However, cardiac tamponade as the initial disease presentation is exceedingly uncommon.

## Case presentation

An 18-year-old male with a past history of gastroesophageal reflux disease (GERD) presented to the emergency department (ED) with one day of burning substernal chest pain and vital signs which were within normal limits. Physical examination was without focal findings. The basic metabolic panel (BMP) and complete blood count (CBC) were within normal limits, single troponin was undetectable, and electrocardiogram (ECG) and chest x-ray did not reveal any acute pathology. A clinical diagnosis of gastritis was made based on recent alcohol ingestion, multiple episodes of emesis, and significant symptom improvement with a gastrointestinal (GI) cocktail containing viscous lidocaine, simethicone aluminum hydroxide, and magnesium hydroxide. The patient was discharged with a proton pump inhibitor (PPI) prescription.

**Figure 1:**
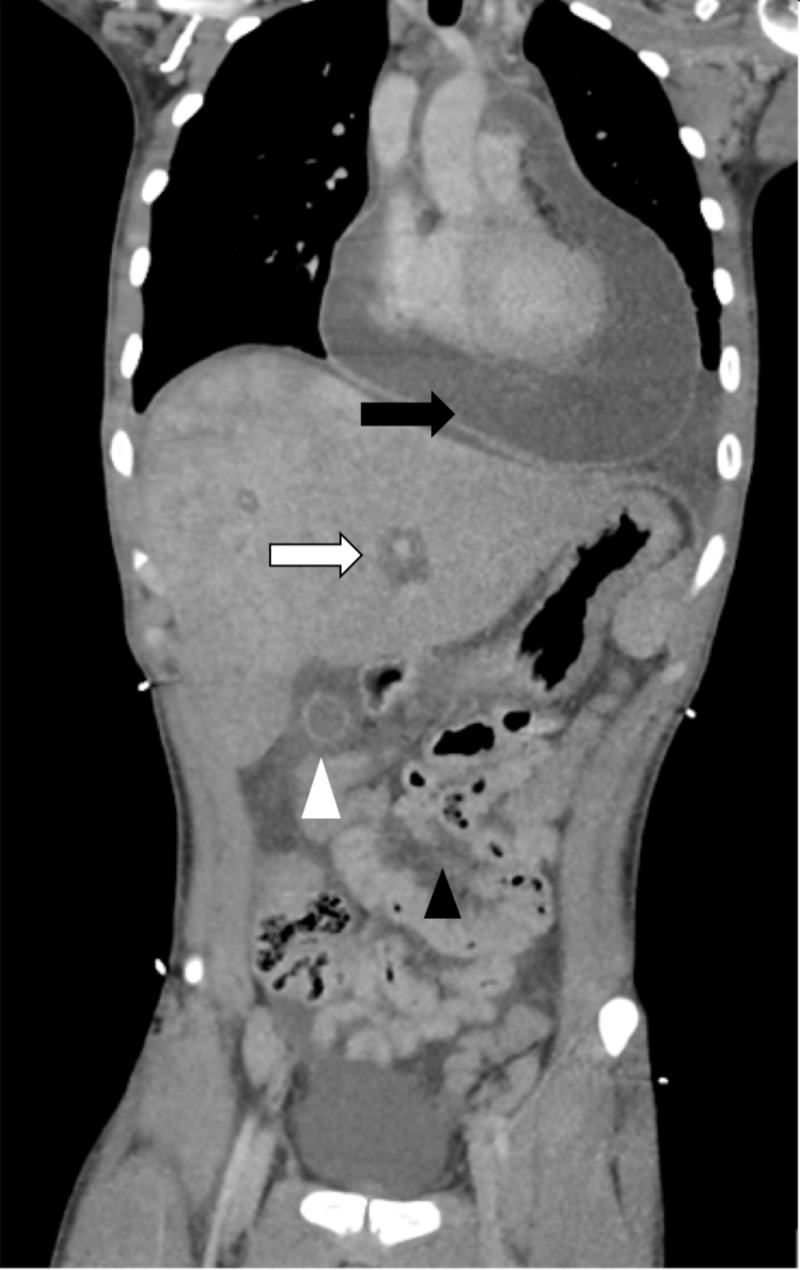
Computed tomography (CT) imaging showing the pericardial effusion (black arrow), ascites (black arrowhead), hepatic congestion (white arrow), and gallbladder wall thickening (white arrowhead)

**Figure 2:**
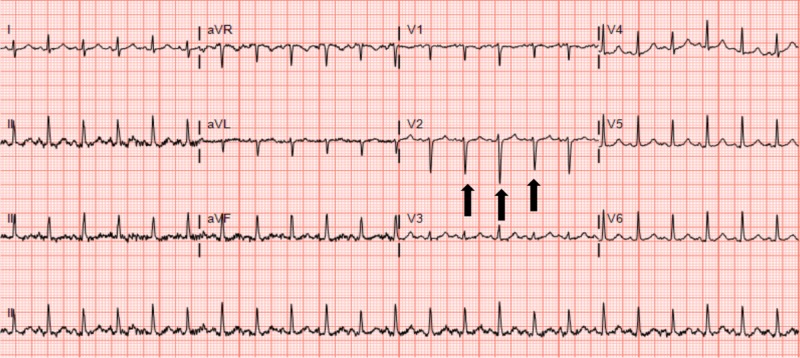
Electrocardiogram (ECG) revealing sinus tachycardia and electrical alternans

Ten days later, the patient returned to the ED with two days of hematochezia with abdominal pain and non-bloody emesis. Initial vital signs showed a heart rate of 141 beats per minute and oxygen saturation of 90% on room air. Intravenous crystalloid fluid boluses and supplemental oxygen were administered for suspicion of hypovolemia due to the gastrointestinal pathology, with a resulting heart rate of 133 beats per minute, oxygen saturation of 94%, and blood pressure of 113/64 millimeters of mercury. Physical examination revealed a young male in acute distress, speaking in brief phrases with diffuse abdominal tenderness. A digital rectal examination was without gross blood. With hemodynamic and mild clinical improvement and the suspicion of gastrointestinal etiology accounting for his symptoms, the patient underwent computed tomography (CT) imaging. A CT scan of the abdomen and pelvis with subsequent chest extension revealed a pericardial effusion up to 4 cm with diffuse ascites, hepatic congestion, and gallbladder wall thickening (Figure 1). Bedside cardiac ultrasound confirmed a large effusion with evidence of right ventricular collapse supporting a diagnosis of cardiac tamponade (Video 1). ECG revealed sinus tachycardia with electrical alternans (Figure 2). Continued large volume crystalloid boluses were given for pre-load support. The cardiology team emergently took the patient to the catheterization lab, where pericardiocentesis drained approximately 1,500 milliliters of serosanguinous fluid. Laboratories drawn prior to this procedure revealed lactic acidosis (lactate - 8.71 millimoles per liter, pH - 7.18, anion gap - 28 milliequivalents per liter), acute kidney injury (blood urea nitrogen - 25.1 milligrams per deciliter, creatinine - 1.94 milligrams per deciliter), and transaminitis (aspartate aminotransferase - 379 units per liter, alanine aminotransferase - 266 units per liter), indicating decreased systemic perfusion. Post-procedure, the patient was admitted to the intensive care unit where he shortly developed a fever of 38.9℃ (102.1°F), coagulopathy (prothrombin time - 33.3 seconds, activated partial thromboplastin time - 43.2 seconds), and fulminant liver failure (aspartate aminotransferase > 7,000 units per liter, alanine aminotransferase - 4,141 units per liter, albumin - 2.6 grams per deciliter), suspected to be due to ischemic hepatitis (shock liver). Acute viral and bacterial workup for the source of the pericardial pathology was unrevealing.

**Video 1:** Bedside cardiac ultrasound revealing a large pericardial effusion and right ventricular collapse suggestive of tamponade

The patient eventually fully recovered, and further laboratory evaluation revealed positive antinuclear antibody (ANA), anti-double-stranded deoxyribonucleic acid (anti-dsDNA), and low C3 and C4 complement levels. Coupled with an exudative, aseptic pericardial effusion, the patient met 2012 SLICC Criteria for a diagnosis of SLE [2]. Further history obtained as an outpatient revealed recent arthralgias on review of systems and a paternal great grandmother diagnosed with SLE. The patient was placed on steroids and hydroxychloroquine, with noted symptom improvement and without further hospital admission to date.

## Discussion

Cardiac tamponade is a potentially lethal consequence of pericardial effusion, as it may lead to impaired diastolic and systolic pump function, obstructive shock, and complete cardiovascular collapse if not promptly treated. With tamponade only occurring in up to 1% of SLE patients, this sequela is an uncommon occurrence. Common presenting symptoms of SLE include fatigue, joint pain, and fevers [5]. Nonspecific symptoms such as these, compounded by disease heterogeneity, may lead to delayed diagnosis. Studies have reported the average interval time from initial symptom onset to formal diagnosis ranging from six months to four years [6]. However, sparse reports of cardiac tamponade as the initial presentation of SLE have been reported [4, 7-13]. This current case differs from these previously cited cases, including patient sex and age, volume and rapidity of pericardial effusion, and subsequent development of tamponade development. Males account for 4% - 22% of patients with SLE [1]. Our patient’s age of presentation is modestly outside the mean age range of both male symptom onset and diagnosis in SLE (26 - 38.4 years and 26 - 55 years, respectively), adding to the case’s novelty [14]. Only two of the reported cases above had affected patients of the male sex, and the ages of those male patients, 32 and 61 years of age, are significantly older than that of our patient [7, 13]. While even small volume pericardial effusions may lead to tamponade, there is a correlation of larger volumes being predictive of this sequela [15]. Of the cited cases from our literature search, the 1,500 milliliters of pericardial fluid drained from our patient was of significantly higher volume than those of other cases, with the next closest volume reported being 1,000 milliliters [10]. Moreover, as the patient presented only 10 days earlier with documented relatively unremarkable chest x-ray and electrocardiogram, as well as an unremarkable outpatient basic laboratory workup just two days prior to tamponade presentation, these measures serve as excellent baseline markers to demonstrate the rapid onset of his condition. The other cases reported did not report similar documented imaging or labs to establish a suggested timeframe in which the effusion and tamponade may have developed. Retrospectively, the initial presentation to the emergency department for chest pain 10 days earlier was likely the initial onset of pericarditis, despite the absence of commonly associated ECG findings. As some studies have found, males with SLE have a higher frequency of serositis, and pericardial effusions may present relatively early in the disease course as well [16].

While the diagnosis is challenging, multiple studies have demonstrated that SLE is a condition in which early diagnosis and treatment are associated with substantial improvements in patient outcomes [17-18]. Cardiac tamponade, an immediately life-threatening condition, is a recognized sequela of this disease, and even symptomatic pericardial effusions are predictive of decreased survival rates, further emphasizing the need for early diagnosis [16].

## Conclusions

SLE is a multisystem, autoimmune, inflammatory condition of extremely variable presentation and severity. Cardiac tamponade is a rare, but lethal, consequence of pericardial effusion secondary to SLE. While young to middle-aged adult minority women with constitutional, cutaneous, musculoskeletal, and renal manifestations are often thought of as classic patients for SLE, the patient presented was a previously healthy 18-year-old male with a seemingly unrelated chief complaint of hematochezia and abdominal pain, necessitating emergent evacuation of massive pericardial effusion and tamponade. This acute presentation displayed a stark contrast from the typical patient and an often indolent disease course.
